# Risk Factors for Loss to Follow-Up among People Who Inject Drugs in a Risk Reduction Program at Karachi, Pakistan. A Case-Cohort Study

**DOI:** 10.1371/journal.pone.0147912

**Published:** 2016-02-03

**Authors:** Rab Nawaz Samo, Ajmal Agha, Sharaf Ali Shah, Arshad Altaf, Ashraf Memon, Meridith Blevins, Han-Zhu Qian, Sten H. Vermund

**Affiliations:** 1 Polio Eradication Initiative, World Health Organization, Larkana, Pakistan; 2 Department of Community Health Sciences, Aga Khan University, Karachi, Pakistan; 3 Bridge Consultants Foundation, Karachi, Pakistan; 4 Sindh AIDS Control Program, Karachi, Pakistan; 5 Vanderbilt Institute for Global Health & Department of Biostatistics, Vanderbilt University School of Medicine, Nashville, Tennessee, United States of America; 6 Vanderbilt Institute for Global Health & Department of Medicine, Vanderbilt University School of Medicine, Nashville, Tennessee, United States of America; 7 Vanderbilt Institute for Global Health & Department of Pediatrics, Vanderbilt University School of Medicine, Nashville, Tennessee, United States of America; UCL Institute of Child Health, University College London, UNITED KINGDOM

## Abstract

**Introduction:**

Retention of male people who inject drugs (PWIDs) is a major challenge for harm reduction programs that include sterile needle/syringe exchange in resource-limited settings like Pakistan. We assessed the risk factors for loss to follow-up among male PWIDs enrolled in a risk reduction program in Karachi, Pakistan.

**Methods:**

We conducted a prospective cohort study among 636 HIV-uninfected male PWIDs enrolled during March-June 2009 in a harm reduction program for the estimation of incidence rate. At 24 months post-enrollment, clients who had dropped out of the program were defined as lost to follow-up and included as cases for case-cohort study.

**Results:**

The median age of the participants was 29 years (interquartile range: 23–36). Active outreach accounted for 76% (483/636) of cohort recruits. Loss to follow-up at 24 months was 25.5% (162/636). In multivariable logistic regression, younger age (AOR: 0.97, 95% CI: 0.92–0.99, *p* = *0*.*028*), clients from other provinces than Sindh (AOR: 1.49, 95% CI: 1.01–2.22, *p* = *0*.*046*), having no formal education (AOR: 3.44, 95% CI: 2.35–4.90, *p<0*.*001*), a history of incarceration (AOR: 1.68, 95% CI: 1.14–2.46, *p<0*.*008*), and being homeless (AOR: 1.47, 95% CI: 1.00–2.19, *p<0*.*049*) were associated with loss to follow-up.

**Conclusions:**

Our cohort retained 74.5% of male PWIDs in Karachi for 24 months. Its loss to follow up rate suggested substantial ongoing programmatic challenges. Programmatic enhancements are needed for the highest risk male PWIDs, i.e., younger men, men not from Sindh Province, men who are poorly educated, formerly incarcerated, and/or homeless.

## Introduction

In 1987, a Pakistani national was diagnosed with human immunodeficiency virus/acquired immune deficiency syndrome (HIV/AIDS) for the first time [1, 2]. The HIV epidemic in Pakistan follows what has been termed the Asian epidemic model [3] and has been driven by injection drug use (IDU). Until 2003, Pakistan’s HIV epidemic was nascent, with very low level prevalence documented in key populations, including people who inject drugs (PWIDs), male sex workers (MSW), female sex workers (FSW), long distance truckers, and *hijra* sex workers (transgender and/or transvestite). In 2004; the HIV epidemic in PWIDs grew quickly such that Pakistan evolved from a nascent to a concentrated HIV epidemic when 9.3% of injection drug users tested were detected HIV seropositive in the city of Larkana, Sindh Province [4, 5]. The HIV epidemic expanded dramatically among PWIDs to 23% in 2006 and 30% in 2007 in the mega-city of Karachi [5, 6]. In 2008; 50% of PWIDs surveyed at Sargodha were found infected with HIV [7].

The government of Pakistan established the Federal Committee on AIDS (FCA) in 1987 [8],as well as a National AIDS Control Program (NACP) in the Ministry of Health for HIV prevention and control [9, 10]. The initial focus was testing and laboratory-based services nationwide supported by World Health Organization [11]. The scope of the services provided by NACP was extended and provincial AIDS control programs were established in 1993 in order to reassign the task of direct HIV control and prevention to the provinces [11]. In July 2002 the program was transformed into the 'Enhanced National AIDS Control Program' with a broader package of services, though opiate substitution therapy was still unavailable [12].

In 2012, the federal HIV/AIDS control program was almost fully devolved to the provinces as a consequence of the 18^th^ Amendment to Pakistan’s Constitution [13, 14]. In response to the growing HIV epidemic, a countrywide network of drop-in centers (DICs) was established in the major cities of Pakistan for comprehensive HIV prevention among PWIDs offering community-based outreach services like needle exchange, condoms for safer sex, and testing and counseling for HIV. Yet the country is lacking in offering modern opiate substitution treatment, as well as Methadone and Buprenorphine therapy. Other harm reduction services in Pakistan were started with the support of 'Program Acceleration Funds' in seven major cities with the support of the U.K. Department for International Development (DFID) and United Nations Office of Drugs and Crime (UNODC) [15].

Experts have estimated that Pakistan has 141,000 PWIDs [16]. Our experience shows that retention of PWIDs in risk reduction programs poses a substantial challenge to the implementers and stakeholders of the program in the resource-limited setting of Pakistan. Many clients do not adhere to the services of the programs due to social, economic, and political factors, including stigma, incarceration, and internal migration. Within the context of a study of HIV seroincidence among PWIDs in Karachi, we assessed risk factors for loss to follow-up.

## Materials and Methods

### Study setting and participants

We conducted a cohort study in Karachi for the estimation of incident rate of HIV among male PWIDs and risk factors associated with loss to follow up. Male PWIDs attending three drop in centers providing needle exchange services were recruited. A cohort of 636 male PWIDs was enrolled from March to June, 2009 and followed for two years. The study design and the HIV incidence rate (12.4 per 100 person-years) were described previously [17]. At the end of the study; a total of 474 (74.5%) were retained with the program while 162 (22.5%) PWIDs were lost to follow up. We took 162 PWIDs who were loss to follow up as cases and took all 474 (74.5%) PWIDs retained with the program as controls for case-cohort study for the estimation of risk factors associated with loss to follow up.

### Cohort follow-up

Male PWIDs were asked to visit drop-in centers daily to exchange used needles and syringes for new ones, and for receiving risk reduction counseling. Study participants were followed by the staff of the program (outreach workers) on a daily basis while the local researchers followed on monthly basis. Male PWIDs who dropped out before the two year follow-up period were defined as lost to follow-up and taken as cases for case-cohort study.

### Data collection

Data were collected by research-trained interviewers working with the harm reduction program at three drop-in centers. A questionnaire adapted from the voluntary counseling and testing (VCT) form was administered once at the time of enrollment of male PWIDs into the program. The questionnaire was piloted prior to the administration to the participants for improvement in clarity and likely validity. The questionnaire included demographic and personal history, pattern of drug use, injection-related information, health-seeking, sexual behavior, and knowledge about HIV/AIDS. The study was approved by Ethics Review Board of the Aga Khan University. Privacy and confidentiality of the participants was assured at all levels of the study, including password protection of all data. Written informed consent was obtained from eligible study participants at the enrollment. The eligibility criteria included male PWIDs registered with harm reduction program and residing in Karachi with HIV negative sero status at the time of enrollment with sound health to understand the study purpose and furnish the consent for the study.

The study participants had the right to refuse blood tests, interviews and withdrawal from the study at any time without compromising their access to the services of the drop-in centers. No monetary incentive was offered for participation in the study.

### Statistical analysis

The data were double-entered into EpiInfo^®^ version 6.0 (Centers for Disease Control and Prevention, Atlanta, Georgia, USA) using two separate data-entry operators. Inconsistencies were reconciled via questionnaire examination and re-interview, whenever possible. Data were analyzed using the Statistical Package for Social Sciences (SPSS^®^) version 17 (IBM SPSS, Inc., Chicago, Illinois, USA). We calculated median and interquartile range for continuous variables and proportions for categorical variables. The experience of our stakeholders of the program showed that age, residence near to the service providing center, level of education, marital status, history of incarceration, having home and family have significant role in perception of the disease and prevention. Owing to importance of these a priori variables were included in the logistic regression model. Using logistic regression, we estimated crude odds ratios (COR) and 95% confidence intervals (95% CI). For multivariable modeling and estimation of adjusted odds ratio (AOR), all variables deemed important by prior hypothesis as well as variables having *p≤0*.*2* in univariable analyses were included [18].

## Results

### Demographics and drug use behaviors of study participants

The median age of the participants was 29 years (interquartile range: 23–36). Active outreach accounted for 483 (76%) for recruitment and registration of clients. Most participants 567 (89.2%) were Muslims, though Christians were heavily overrepresented compared to their proportion in the general population. Over 40% participants were from provinces other than Sindh Province. Nearly two-thirds (64.9%) had no formal education and over half (55%) were unmarried. The most common venue for injecting drugs was the street 538 (84.6%). Peer pressure (59.1%) was cited as the major reason for current drug use. Half of the participants (49.8%) had a history of incarceration (Table 1).

**Table 1:** Characteristics of 636 men who inject drugs in a Karachi cohort: 2009 enrollment data and 2-year follow-up status.

Characteristics	Loss to follow-up	Retained	Total
	n (%)	n (%)	n (%)
Overall	162 (22.5)	474 (74.5)	636 (100%)
Age (year), median (IQR^a^)	30 (23–39)	28 (23–35)	29 (23–36)
**Source of registration**			
Outreach	122 (75.3)	361 (76.2)	483 (76.0)
Other^b^	40 (24.7)	113 (23.8)	153 (24.0)
**Religion**			
Muslim	148 (91.4)	419 (88.4)	567 (89.2)
Non Muslim	14 (8.6)	55 (11.6)	69 (10.8)
**Residence**			
Sindh province	109 (67.3)	255 (53.8)	364 (57.2)
Other than Sindh^c^	53 (32.7)	219 (46.2)	272 (42.8)
**Education**			
Formal education	93 (57.4)	130 (27.4)	223 (35.1)
No formal education	69 (42.6)	344 (72.6)	413 (64.9)
**Currently employed**			
Yes	71 (43.8)	231 (48.7)	302 (47.5)
No	91 (56.2)	243 (51.3)	334 (52.5)
**Marital status**			
Married	66 (40.7)	220 (46.4)	286 (45.0)
Unmarried	96 (59.3)	254 (53.6)	350 (55.0)
**Having a home**			
Yes	108 (66.7)	271 (57.20)	379 (59.6)
No	54 (33.3)	203 (42.8)	257 (40.4)
**Ever history of incarceration**			
Yes	65 (40.1)	252 (53.2)	317 (49.8)
No	97 (59.9)	222 (46.8)	319 (50.2)
**Principal place of drug use**			
Street	133 (82.1)	405 (85.4)	538 (84.6)
Home	29 (17.9)	69 (14.6)	98 (15.4)
**Family history of injection drug use**			
Yes	10 (6.2)	81 (17.1)	91 (14.3)
No	152 (93.8)	393 (82.9)	545 (85.7)
**Median age in years, 1**^**st**^ **drug use (IQR)**	15 (13–18)	16 (13–19)	16 (13–19)
**Main reason reported for current drug use**			
Peer Pressure	79 (48.8)	297 (62.7)	376 (59.1)
Emotional Pain Relief	19 (11.7)	49 (10.3)	68 (10.7)
Sex enjoyment	22 (13.6)	41 (8.6)	63 (9.9)
Pleasure	19 (11.7)	35 (7.4)	54 (8.5)
Physical Pain Relief	16 (9.9)	29 (6.1)	45 (7.1)
Curiosity	3 (1.9)	16 (3.4)	19 (3.0)
Escape^d^	4 (2.5)	7 (1.5)	11 (1.7)

^a^IQR, interquartile range.

^b^Government organization, non-governmental organization, community, and friends

^c^Punjab, Baluchistan, Khyber Pakhtunkhwa, and Kashmir

^d^Escape refers to flight from social pressures.

### Risk factors for loss to follow-up

The 2-year rate of loss to follow-up was 25.5%, an average of nearly 13% per year. Six factors having p = ≤0.2 in uni variable analysis; lower age (*p = 0*.*110*), coming from a province other than Sindh (*p*<*0*.*001*), having no formal education (*p*<*0*.*001*), being unmarried (*p = 0*.*201*), history of incarceration (*p* = *0*.*004*), and homelessness (*p* = *0*.*034*) were selected for multivariable modeling (Table 2).

**Table 2:** Crude and adjusted odds ratios of risk factors for loss to follow-up at 24 months among men who inject drugs receiving harm reduction services in Karachi (636).

Characteristics	COR	(95% CI)	P-value	AOR	(95% CI)	P-value
Age (per 1 year increase)	0.98	(0.96, 1.00)	*0.114*	0.97	(0.92, 0.99)	0.028
**Residence**						
Other than Sindh^a^ vs. Sindh province	3.62	(2.41, 5.20)	*<0*.*001*	1.49	(1.01, 2.22)	0.046
**Education**						
No formal education vs. Formal education	3.56	(2.46, 5.16)	<0.001	3.44	(2.35, 4.90)	<0.001
**Marital status**						
Unmarried vs. Married	0.70	(0.55, 1.14)	*0*.*201*	0.72	(0.48, 1.08)	0.117
**Ever history of incarceration**						
Yes vs. No	1.69	(1.17, 2.43)	*0*.*004*	1.68	(1.14, 2.46)	0.008
**Have a home**						
No vs. yes	1.49	(1.03, 2.17)	*0*.*034*	1.47	(1.00, 2.19)	0.049

Odds ratio obtained from logistic regression. PWIDs, people who inject drugs; COR, crude odds ratio; AOR, adjusted odds ratio; 95% CI, 95% confidence interval

^a^Punjab, Baluchistan, Khyber Pakhtunkhwa, Bengal, Kashmir

In multivariable analysis, five factors remained as significant predictors for loss to follow-up (Table 2): younger age (AOR for each year of age: 0.97, 95% CI: 0.95–0.99, *p* = *0*.*028*), coming from a province other than Sindh (AOR: 1.49, 95% CI: 1.0–2.22, *p*<*0*.*046*), having no formal education (AOR: 3.44, 95% CI: 2.35–5.03, *p* <*0*.*001*), prior incarceration (AOR: 1.68, 95% CI: 1.14–2.46, *p*<*0*.*008*), and homelessness (AOR: 1.47, 95% CI: 1.00–2.19, *p* = *0*.*0495*).

## Model Adequacy

The model adequacy was assessed by Hosmer & Lameshow goodnes of fit test. The model adequacy showed that model is fit accurately (Chi sq: 4.65, df: 8, *p* = *0*.*794*).

## Discussion

Despite the urgency and magnitude of the HIV epidemic among male PWIDs in Pakistan, this is the first ever study reporting risk factors for loss to follow-up among clients of HIV risk reduction program in Pakistan. The loss to follow-up of male PWIDs in the program over two years was 25.5%. The HIV incidence rate was 12.4 per 100 person years for men who were retained in the program suggests that the true HIV incidence rate may be higher than measured in our study [17].

Younger age (*p = 0*.*028*), origin outside of Sindh province (*p = 0*.*046*), having no formal education (*p<0*.*001*), ever history of incarceration by law enforcing agencies (*p = 0*.*008*), and having no home (*p = 0*.*049*) were significant predictors of loss to follow-up in the program at enrollment. There are many studies suggesting that adolescents and young adults are at a higher risk of non-adherence to HIV care and loss to follow-up [19–22]. Mobility may be high for younger PWIDs, they may have fewer sources of money for drugs or food, and they may not appreciate the value of drop-in center services (needle exchange, condoms, and health) as much as older PWIDs.

We think that origin outside of Sindh province as a risk factor for loss to follow-up reflects the propensity of drug users to migrate; persons may have left Karachi to go to their respective provinces and native towns. The lack of any formal education was common and was also a risk for loss to follow-up. Persons with some education might have more understanding about the disease risks inherent in drug injection and the potential beneficial features of the drop-in program. Prior incarceration among injection drug users is typically attributed to theft and/or to drug use itself. Male PWIDs with such a prior experience may be especially mobile in an effort to evade from law enforcement agencies. Homelessness is another logical risk factor for loss to follow-up. Clients enrolled in the drop-in program that live nearby can access services with less difficulty than persons living on the streets who may have trouble in adhering to a fixed site program. Many of these risk factors are strongly linked to low self-esteem, low social support, perceived stigma, and poor coping skills in the context of chaotic lifestyles linked to opiate addiction [23–45].

The retention of male PWIDs either in needle exchange programs or even in research cohorts followed for incidence or intervention is a challenge as reported by other Asian studies, though some clinical trials and risk reduction programs have achieved higher rates of retention and/or adherence [46–60]. The risk factors that we identified were not surprising, but nonetheless help programs that target those men who are more likely to be lost to follow-up early in their program engagement.

Our cohort’s 74.5% retention rate of male PWIDs in risk reduction program was achieved with a tiny research budget and strong support from existing risk reduction programs. For a small “top-up” of their salaries, we were able to use existing program staff to help us with questionnaires and outreach. Other reported retention rates of injection drug users have also been high, e.g., 80% in St Petersburg, Russia and 70% in Sichuan province, China [16]. The retention rate of male PWIDs in our study sample indicates that the quality of services given to the clients is reasonably attractive to the clients. Future improvements in service delivery might further improve the retention rate, ultimately decreasing the risk of HIV transmission and disease burden at a national level. Many more drop-in centers are needed in Karachi to meet demand and the number of mobile service units for needle exchange services should be expanded to mitigate the burden of HIV, hepatitis C, hepatitis B, and non-infectious morbidity in PWIDs in Pakistan [61–63]. Other Asian programs also suggest the challenges but ultimately value opiate agonist substitution therapy to reduce needle use and increase program retention [64–76]. It is tragic that such services are not available to PWIDs in Pakistan, enabling them to reduce and even eliminate needle/syringe use as a start to a healthier lifestyle.

Our study has both strengths and limitations. This is the first-ever study conducted in Pakistan for the determination of risk factors for loss to follow-up among male PWIDs enrolled in a risk reduction program. That we can now better understand individuals more likely to drop-out can empower outreach for this key population. Since our study was conducted in three drop-in centers, results can neither be generalized to all male PWIDs in drop-in centers, nor to PWIDs not participating in risk reduction programs. Data obtained at enrollment were self-reported by the cohort participants; so information bias, social response bias, and recall bias are all possible. Dates of loss to follow-up and updated client information were not collected (data collection was at time zero and two years) such that the data could not be analyzed as time to event with time updated exposures. While clients from three drop-in centers were interviewed, the center was not recorded in the database which does not allow for center-specific estimates and covariate adjustment.

While the loss to follow-up among male PWIDs in risk reduction programs in Karachi was not as high as we expected, we still lost a quarter of cohort members by 24 months follow-up. We recommend four interventions: 1) Drop-in programs can identify likely future program drop-outs at the time of program entry, adapting services to maximize retention; 2) Many more drop-in centers and mobile needle exchange programs are needed to serve PWIDs and reduce the risk of blood-borne infections [77, 78]; 3) Further improvements in service delivery would surely improve the retention rates, especially opiate substitution therapy using Buprenorphine for its simplicity and lower cost; 4) Primary prevention of drug abuse should include expanded school and job training opportunities, given the limited resource setting of Pakistan. While our socio demographic risk factors were not surprising, their recognition can nonetheless help programs target those men who are more likely to be lost to follow-up early in their program engagement.
